# Characterization of the SARS-CoV-2 genomes in Egypt in first and second waves of infection

**DOI:** 10.1038/s41598-021-99014-4

**Published:** 2021-11-03

**Authors:** Abdel-Rahman N. Zekri, Abeer A. Bahnasy, Mohamed M. Hafez, Zeinab K. Hassan, Ola S. Ahmed, Hany K. Soliman, Enas R. El-Sisi, Mona H. Salah El Dine, May S. Solimane, Lamyaa S. Abdel Latife, Mohamed G. Seadawy, Ahmed S. Elsafty, Mohamed Abouelhoda

**Affiliations:** 1 Cancer Biology Department, Virology and Immunology Unit, National Cancer Institute, Cairo University, Cairo, 11796 Egypt; 2 Surgical Pathology Department, National Cancer Institute, Cairo University, Cairo, 11796 Egypt; 3 Clinical and Chemical Pathology Department, Faculty of Medicine, Cairo University, Cairo, Egypt; 4 Main Chemical Laboratories, Egypt Army, Cairo, Egypt; 5 Systems and Biomedical Engineering Department, Faculty of Engineering, Cairo University, Cairo, 12613 Egypt

**Keywords:** Biochemistry, Molecular biology

## Abstract

At Wuhan, in December 2019, the SRAS-CoV-2 outbreak was detected and it has been the pandemic worldwide. This study aims to investigate the mutations in sequence of the SARS-CoV-2 genome and characterize the mutation patterns in Egyptian COVID-19 patients during different waves of infection. The samples were collected from 250 COVID-19 patients and the whole genome sequencing was conducted using Next Generation Sequencing. The viral sequence analysis showed 1115 different genome from all Egyptian samples in the second wave mutations including 613 missense mutations, 431 synonymous mutations, 25 upstream gene mutations, 24 downstream gene mutations, 10 frame-shift deletions, and 6 stop gained mutation. The Egyptian genomic strains sequenced in second wave of infection are different to that of the first wave. We observe a shift of lineage prevalence from the strain B.1 to B.1.1.1. Only one case was of the new English B.1.1.7. Few samples have one or two mutations of interest from the Brazil and South Africa isolates. New clade 20B appear by March 2020 and 20D appear by May 2020 till January 2021.

## Introduction

Severe acute respiratory syndrome coronavirus-2 (SARS-CoV-2) was first detected in late December 2019 as an etiological agent for pneumonia cluster cases in Wuhan City, Hubei Province, China^1–3^. The disease caused by the infection of this new pathogen is called Coronavirus 2019 disease (COVID-19) and has spread rapidly. A pandemic has been reported by the World Health Organization (WHO) and it has affected almost every country worldwide. By 12 February 2021, more than 107 million confirmed individual infections and more than 2 million confirmed deaths have been reported. The ability to transmit prior to becoming symptomatic is one of the reasons for its rapid spread^4^.

SARS-CoV-2 airborne transmission seems likely to occur primarily through respiratory droplets and physical contact between humans beings^7,8^. The period of incubation ranges from 2 to 14 days; however, longer intervals were reported^9^. SARS-CoV2 infections are common with a wide variety of healthcare procedures, including asymptomatic and fatal, and are often undiagnosed with low to moderate symptoms including sore throat, dry cough, and fever^5,6^.

SARS-CoV-2 belongs to the Order *Nidovirales*, Family *Coronaviridae*, Subfamily *Orthocoronavirinae*, Genus *Betacoronavirus*, Subgenus *Sarbecovirus*, Species *Severe acute respiratory syndrome-related coronavirus* and individuum SARS-CoV-2 with the addition of the strain/sequence, e.g., SARS-CoV-2 Wuhan-Hu-1 as the reference strain^7^. SARS-CoV-2 is enveloped, positive-stranded RNA viruses with about 30 kb genome encoding multiple proteins. The SARS-CoV-2 structure, size (80–120 nm), genome, and RNA-based pathogenesis is resemble those of other coronaviruses^8–11^.

Initial translation of the positive-stranded RNA from virus particles generates a virally encoded replicase enzyme that is necessary for viral replication and generation of sub-genomic viral RNAs (sgRNAs). ORF1ab occupies about two-thirds of the 5′ prime end of the genome. ORF1ab is followed by spike (S), ORF3a, envelope (E), membrane (M), ORF6, ORF7a, ORF7b, ORF8, nucleocapsid (N) and ORF10. S protein promotes attachment to human angiotensin converting enzyme 2 (ACE2) and fusion to host cells during infection. The E protein regulates the virion assembly. M protein is also involved in the assembly and biosynthesis of new virus particles while N protein forms the Ribonucleoprotein complex and has a variety of roles, including improving viral genome transcription^12^. The spike coronavirus spike protein binding domain sequence is the most variable region that is likely to change. A total of six residues of amino acids are suggested to be essential for binding to the human ACE-2 receptor. According to the SARS-CoV2 amino-acid co-ordinates these are residues L455, F486, Q493, S494, N501, and Y505. Of these six residues of SARS-CoV2 five have is likely due to mutations, deletions or insertions in the S1–S2 of the Coronavirus region^9,13–15^.

The polybasic cleavage site (RRAR) in SARS-CoV2 is located at the junction of two Spike subunits, S1 and S2. This polybasic proteolytic cleavage of S glycoprotein is responsible for determining the viral infectivity as well as the host range as to whether the virus can jump across species, e.g. from bats to humans. Proteases (like furin) cleavage sites may have been acquired by recombination of RNA, and its presence in SARS-CoV2 may have been responsible for infecting human cells. Also, this cleavage site may have allowed the CoV bat to jump into humans and thus initiate the outbreak of COVID-19^9,15–17^.

The pathogenic nature and genetic variations of SARS-CoV-2 suggest its high binding affinities for the host cell and competently bypass or block interferon-triggered immune responses of the host cell. In this study, we tried to investigate the mutations in sequence of the SARS-CoV-2 genome and characterize the mutation patterns in Egyptian COVID-19 patients during different waves of infection.

## Results

### Mutations in SARS-CoV2 genomes second wave of infection in Egypt

Mutation analysis shows a total of 1115 unique mutations (synonymous vs non-synonymous ratio = 1.6:1) from all Egyptian SARS-CoV-2 samples compared to the reference Wuhan-Hu-1 sequence (Accession NC_045512). We found that more than half of the mutations were in ORF1ab polyprotein (60.5%). The least number of mutations were related to the ORF6 and ORF8 protein sequences (0.7%) (Table 1). Of the 1115 mutations, there are 613 missense mutation, 431 synonymous mutation, 25 upstream gene mutation, 24 downstream gene mutation, 10 frameshift mutation, 6 stop gained, and 2 conservative in-frame deletion, 2 disruptive in-frame deletion, 1 splice region mutation & synonymous mutation and 1 start lost (Table 1).

**Table 1:** The Number of gene variations in SARS-CoV2 genomes in the second wave of infection via comparison of the 183 whole genomes to the NC_045512.2 genome sequence the.

Genome segment	Missense mutation	Synonymous mutation	Frameshift deletion/in frame del	Other mutation			
				Upstream	downstream	Stop gained	
ORF1ab	344	306	8	14	0	2	674
S	94	57	0	0	25	1	177
E	8	4	1	2	0	0	15
M	9	13	0	1	0	0	23
N (87)	57	26	1	3	0	0	87
ORF8	20	4	3	2	0	3	32
ORF10	5	1	2	0	0	0	8
ORF 3a	48	15	0	0	0	0	63
ORF6	3	2	0	0	0	1	6
ORF7a	16	3	0	1	0	0	20
ORF7b	9	0	0	0	0	1	10
Total	613	431	15	23	25	8	1115

E: envelope protein; M: membrane glycoprotein; N: Nucleocapsid phosphoprotein; ORF: open reading frame; S: spike glycoprotein; SARS-CoV-2: severe acute respiratory syndrome coronavirus 2.

**Figure 1:**
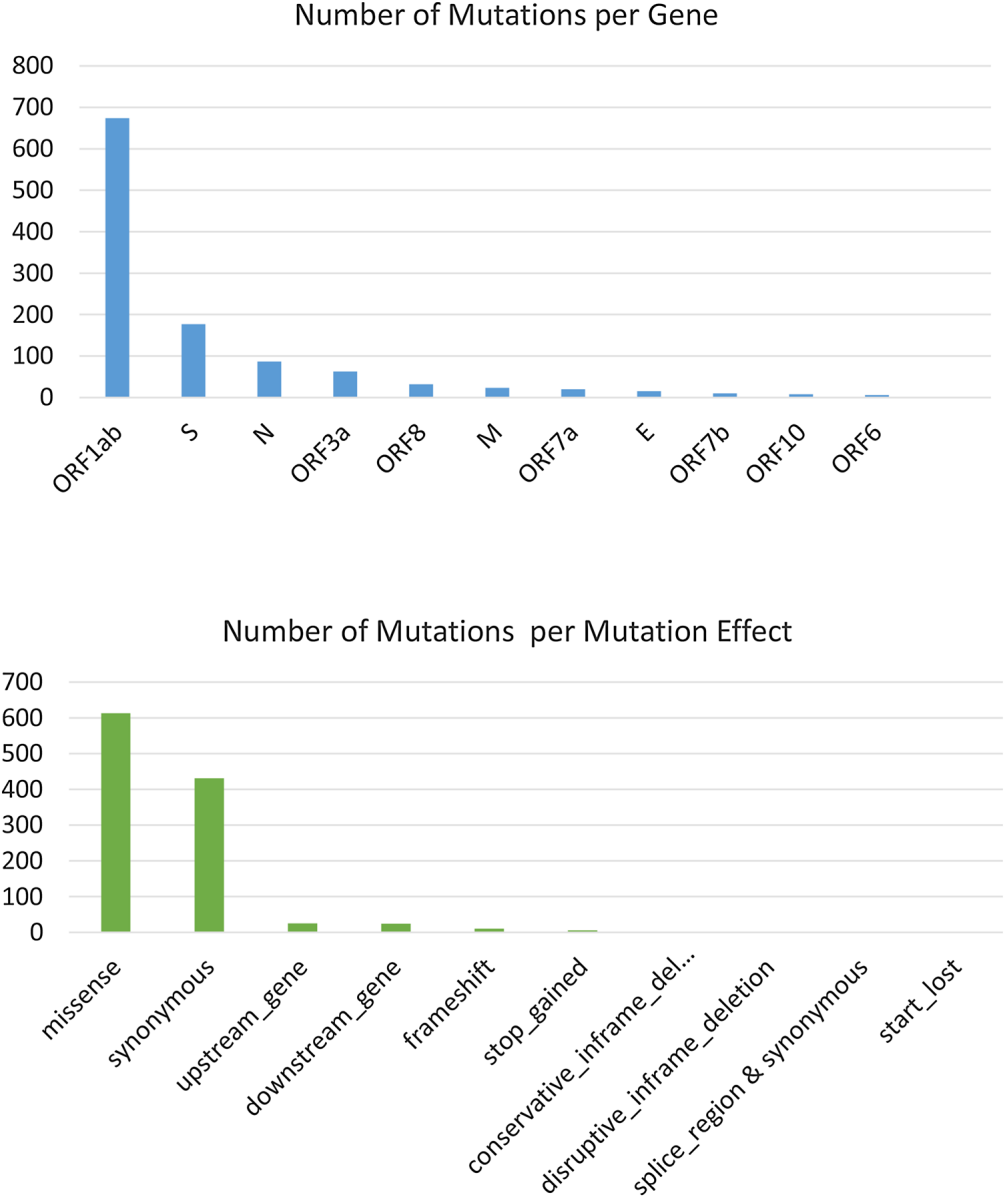
Distribution of the SARS-CoV-2 mutations in the Egyptian sequences. Upper plot includes the number of mutations in each SARS-CoV2 gene. Lower plot incudes the number of mutations in each mutation-effect category.

**Figure 2:**
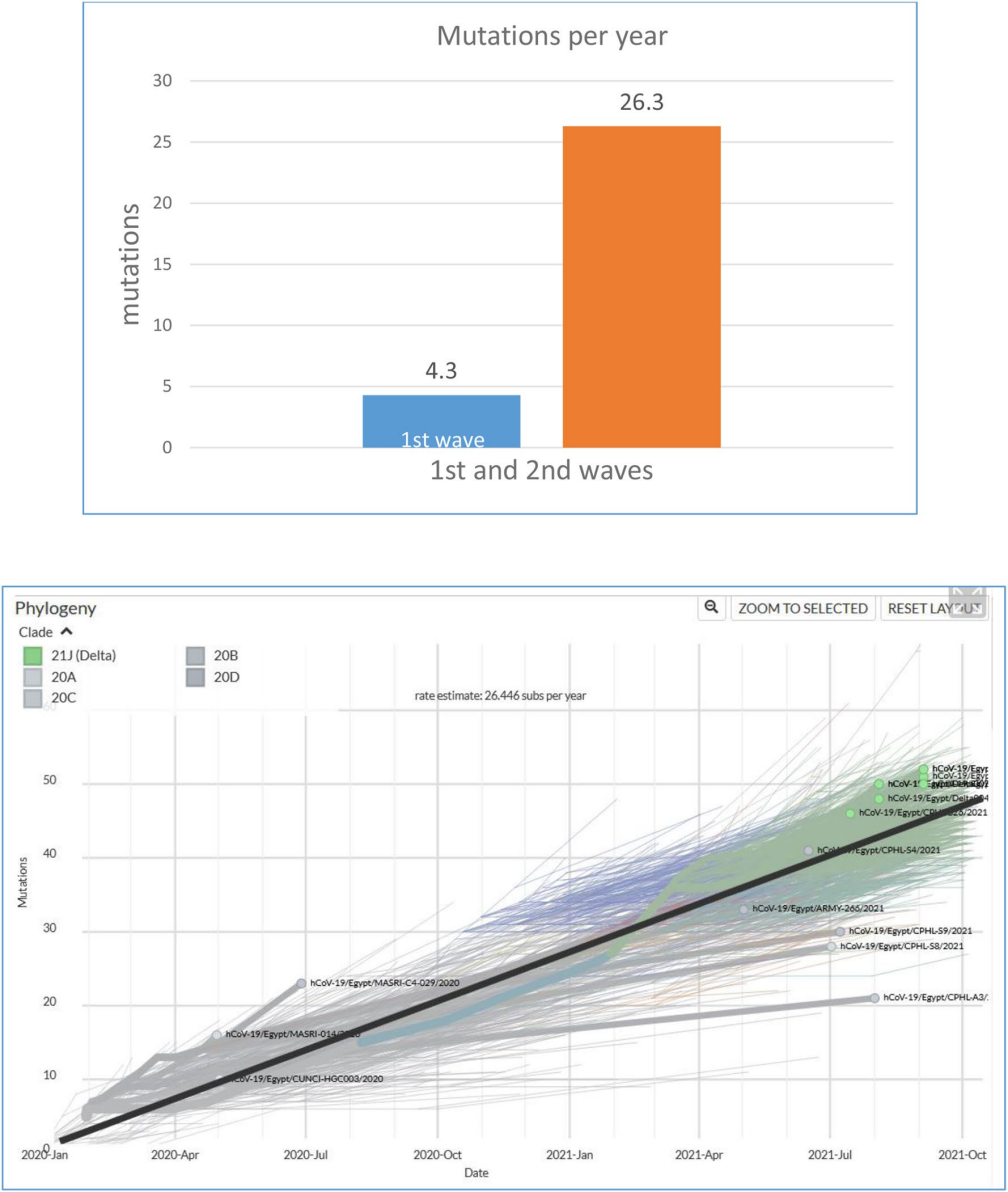
Rate of mutations per sample per year: The upper plot shows the rate of mutations in the Egyptian samples in the first and second waves (1st wave samples were collected between March and April 2020 and 2nd wave samples collected between November and mid-January 2021). The lower plot includes the rate of mutations per sample over different time points in the Egyptian samples (Source: nextstrain.org).

As for their distribution per gene, 674 mutations were found in ORF1ab (60.5%), followed by 177 in S (15.9%), 87 in N (7.8%), 63 in ORF3a (5.7%), 32 in ORF8 (2.9%), 23 in M (2.1%), 20 in ORF7a (1.8%), 15 in E (1.3%), 8 in ORF8 (0.7%) and 6 in ORF6 (0.5%) (Table 1). In comparison to the first wave of infection, there were 204 mutations: 131 in ORF1ab (64%), 30 in S (14.7%), 23 in N, 6 in ORF3a, 6 in ORF7a, 4 in ORF8, 2 in M, 1 in E, and 1 in ORF6 as previously published (Fig. 1 & Supplementary File S1). Additionally, the average number of mutations per sample per year is 26 for Egyptian samples in the second wave, while it was 4 in the first wave. This rare is comparable to the world mutation which is around 22.88 (Fig. 2).

Investigating the frequency of the mutations in the Egyptian samples compared to the world samples, there was no mutation specific to the Egyptian ones in the first and second waves of infection. Tables 2 and 3 include the most frequent mutations in the Egyptian samples.

**Table 2:** The top Frequent Mutations in Egypt and in the world during the second waves.

Position	Ref	Obs	Egy. Freq (n = 183)	Wolrd Freq (n = 371822)	1stWave EgyFreq (n = 265)	Gene	Transcription Pos	AA_change	Type of mutation
23403	A	G	96.17%	93.26%	86.04%	S	c.1841A > G	D614G	Missense mutation
14408	C	T	93.99%	92.86%	61.13%	ORF1ab	c.14144C > T	P4715L	Missense mutation
3037	C	T	92.35%	92.87%	81.51%	ORF1ab	c.2772C > T	F924F	Synonymous mutation
241	C	T	89.07%	90.51%	81.89%	ORF1ab	c.-25C > T	Upstream	Upstream mutation
23731	C	T	72.68%	02.18%	12.83%	S	c.2169C > T	T723T	Synonymous mutation
10097	G	A	71.58%	02.17%	14.72%	ORF1ab	c.9832G > A	G3278S	Missense mutation
13536	C	T	69.95%	01.36%	12.83%	ORF1ab	c.13272C > T	Y4424Y	Synonymous mutation
28908	G	T	68.31%	00.07%	12.45%	N	c.635G > T	G212V	Missense mutation
4002	C	T	67.21%	01.30	12.83%	ORF1ab	c.3737C > T	T1246I	Missense mutation
28881	GGG	AAC	63.39%	34.16%	0.00%	N	c.608_610delGGGinsAAC	RG203KR	Missense mutation
12534	C	T	55.74%	00.15%	12.45%	ORF1ab	c.12269C > T	T4090I	Missense mutation
23593	G	T	55.19%	00.39%	13.58%	S	c.2031G > T	Q677H	Missense mutation

**Table 3:** The top Frequent Mutations in Egypt and in the world during the first waves.

Position	Ref	Obs	Egy. Freq	Wolrd Freq	Gene	Transcription Pos	AA_change	Type of mutation
23403	A	G	98.36%	76.05%	S	c.1841A > G	D614G	Missense mutation
241	C	T	96.72%	74.56%	ORF1ab	c.-25C > T	Upstream	Upstream mutation
3037	C	T	93.44%	75.65%	ORF1ab	c.2772C > T	F924F	Synonymous mutation
14408	C	T	91.80%	75.75%	ORF1ab	c.14144C > T	P4715L	Missense mutation
25563	G	T	49.18%	22.24%	ORF3a	c.171G > T	Q57H	Missense mutation

### Geographical distribution of the SARS-CoV-2 mutations characterizing the variants of interest in Egyptian samples (first and second wave of infection)

We collected the mutations of related to the variants/lineages of interest from the UK B.1.1.7 lineage, B.1.351 South African lineage, the B.1.1.28 Brazilian lineage, US B.1.2 lineage and the 20A.EU1 lineage. 29 of these mutations exist in the Egyptian samples (Table 4) of the second wave. Among these mutations of interest, 18 ones were found in the S protein, where the D614G is the most frequent one. Four mutations of interest were found in the ORF1ab polyprotein, distributed in two regions coding for NSP6 (S367S), and three coded for NSP3 (T1001I),(A1798D) and (S1188L); these come from the England B.1.1.7 and Brazil B.1.1.28 lineages. Three mutations of interest were found in ORF8 (Y73C), (Q27*) and (R52I) coming from England B.1.1.7. Three mutations of interest was observed in N protein (S235F), (T205I) and (D3L), coming from the England B.1.1.7 and South Africa B.1.351 lineages. Two mutations of interest were observed in E protein (V39L) and (P71L), coming from the England B.1.1.7 and South Africa B.1.351 lineages.

**Table 4:** Mutations related to emerging strains in Egyptian Samples.

Pos	Reference	Alternative	EgyFreq	WorldFreq	Gene	AA_Change	Emerging Variants
23403	A	G	96.17%	93.26%	S	D614G	England_B.1.1.7;South_Africa_B.1.3512
2227	C	T	01.09%	22.07%	S	A222V	Spain
21,614	C	T	00.55%	10.12%	S	L18F	Brazil_B.1.1.28
22992	G	A	04.92%	05.53%	S	S477N	Spain
23604	C	A	04.92%	05.24%	S	P681H	England_B.1.1.7
28977	C	T	07.10%	04.99%	N	S235F	England_B.1.1.7
23063	A	T	00.55%	04.98%	S	N501Y	England_B.1.1.7; Brazil_B.1.1.28; South_Africa_B.1.351
11287	GTCTGGTTTT	G	00.55%	04.84%	ORF1ab	S3675	England_B.1.1.7; Spain
3267	C	T	00.55%	04.82%	ORF1ab	T1001I	England_B.1.1.7
24914	G	C	00.55%	04.74%	S	D1118H	England_B.1.1.7
23271	C	A	00.55%	04.74%	S	A570D	England_B.1.1.7
24506	T	G	00.55%	04.73%	S	S982A	England_B.1.1.7
28111	A	G	00.55%	04.73%	ORF8	Y73C	England_B.1.1.7
27972	C	T	01.09%	04.65%	ORF8	Q27*	England_B.1.1.7
28048	G	T	01.09%	04.62%	ORF8	R52I	England_B.1.1.7
5388	C	A	00.55%	04.59%	ORF1ab	A1708D	England_B.1.1.7
25907	G	T	01.09%	02.35%	ORF3a	G172V	US_B.1.2
22879	C	A	00.55%	02.19%	S	N439K	Spain
28887	C	T	07.10%	00.96%	N	T205I	South_Africa_B.1.351
21800	G	T	01.09%	00.55%	S	D80Y	Spain
23593	G	T	55.19%	00.39%	S	Q677H	US_B.1.2
23525	C	T	03.83%	00.35%	S	H655Y	Brazil_B.1.1.28
23012	G	A	00.55%	00.17%	S	E484K	Brazil_B.1.1.28; South_Africa_B.1.351
21974	G	T	00.55%	00.17%	S	D138Y	Brazil_B.1.1.28
21638	C	T	00.55%	00.17%	S	P26S	Brazil_B.1.1.28
3828	C	T	07.65%	00.08%	ORF1ab	S1188L	Brazil_B.1.1.28
26389	G	T	00.55%	00.04%	E	V49L	Brazil_B.1.1.28
26455	CCT	CTT,GTA	03.28%	00.00%	E	P71L	South_Africa_B.1.351
28280	GAT	CTA	00.55	00.00%	N	D3L	England_B.1.1.7

### The D614G and other top frequent mutations

The highest Egyptian frequency mutation in the second wave was found in 176 out of 183 of the viral genome samples. This leads to change in amino acid from aspartic acid (D) to Glycine (G). The D614G amino acid change was found in the spike region of Egyptian strain GR in both the first and the second waves (Tables 2, 3). This amino acid change was accompanied by silent mutation of C241T in a non-coding region, and in C3037T of ORF1a, the missense mutation at C14408T (P214L) in ORF1b.

The most frequent mutation in the second wave of SARS-CoV-2 infection was observed in the first wave of infection. From these top 12 mutations observed in the second wave of infection, there was only one mutation not in the first wave. These mutations included two mutations in S region, two mutations in N region and four mutations in ORF1. Tables 2 and 3 include the most frequent mutations in the Egyptian samples. For both waves of mutations, there was no mutation specific to the Egyptian samples.

The Missense mutation of G28881A, G28882A, and G28883C results in amino acid changes (R202K and G203R) and of G28908T results in amino acid changes G212V in N was observed in the second wave. As shown in Table 2, the spike region contained three nucleotide mutations resulting in three amino acid changes. In addition to the D614G mutation, both of the C23731T mutation and the G23593T mutation in the spike region resulted in amino acid changes T723T and Q677H respectively.

The ORF1ab is transcribed into a multi-protein and subsequently divided into 16 non-structural proteins (NSPs). The Missense mutation of C14408T and synonymous mutation of C13536T resulting in amino acid changes (P4715L and Y4424Y) in RNA-depended-RNA-polymerase region. One synonymous mutation of C3037T resulting in amino acid change F924F in NSP3 region.

### Lineage and phylogenetic analysis

One hundred eighty three whole genome sequences from the second wave of infection and 282 from the first wave of infection with > 99% reads mapped to the reference genome were generated, with average coverage depth of 992 × . All Egyptian whole genome sequences available in GISAID were added to the analysis, making a total of 465 Egyptian sequences.

**Figure 3:**
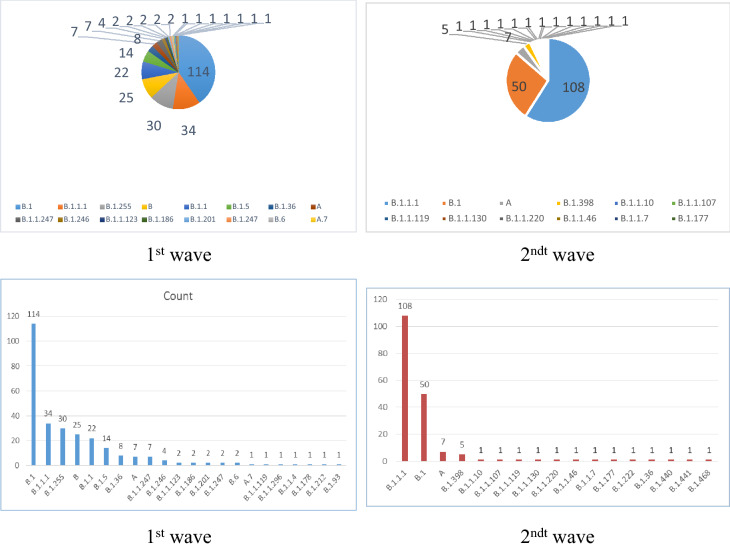
Dominant Lineages/Types of the virus in Egypt during the first and the second waves of infection. Upper plots: Bar charts showing top lineages in the Egyptian samples. Lower plots shows the percentage of lineages. Lineage B.1 represented 40% of cases in the first wave, while lineage B.1.1.1 represented 59% of cases in the second wave.

For the evaluation of lineages, Pangolin (Phylogenetic Assignment of Named Global Outbreak LiNeages) COVID-19 lineage Assigner was used where nearly 22 different lineages was found to be circulating in Egypt and majority of Twenty two lineage groups were identified in the 183 Egyptian sequences of second wave of infection and 17 lineage groups were identified in the 282 Egyptian sequences had infection in the first wave Fig. 3. Lineage B.1 represented 40% of cases in the first wave, while lineage B.1.1.1 represented 59% of cases in the second wave.

Using the Pangolin and Nextstrain methods of lineage classification, isolates in clade 1 were assigned mainly to Pangolin lineage B.1.1.1 and B.1 and Nextstrain clade 20D and 20A. The majority of isolates in Clade 3 belonged to Pangolin lineage A and Nextstrain clade 19B. The Status of the emerging lineages of concerning 1st and 2ndt wave Emerging lineages of concern include: the English (UK) B.1.1.7, (20I/501Y.V1), the South African B.1.351 (20H/501Y.V2), the Brazilian ones B1.1.28, (renamed “P.1”) and the USA B.1.2 (20C-US).

**Figure 4:**
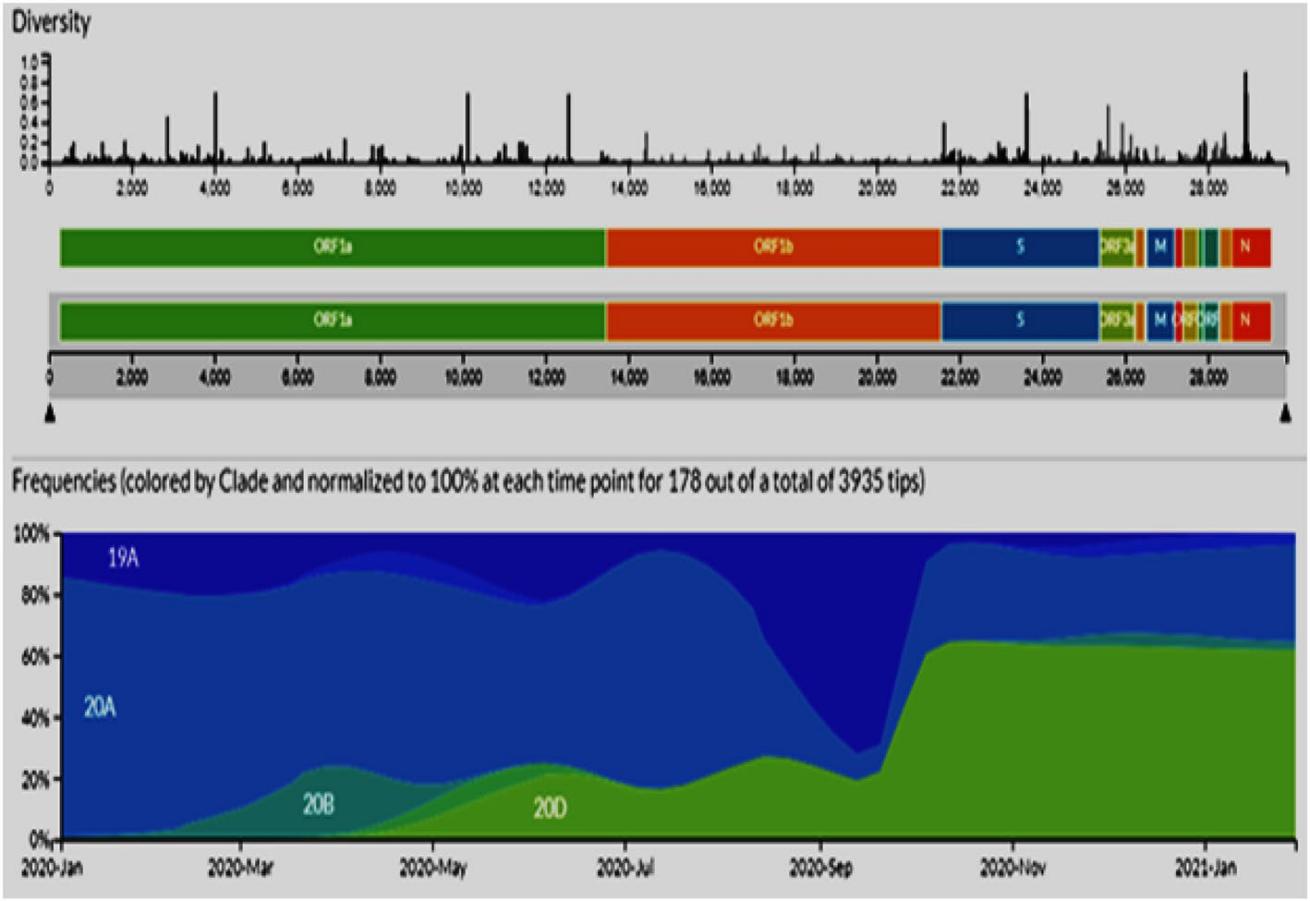
Clade distribution (based on phylogenetic analysis) in second wave of infection in the Egyptian isolates compared to the Global Pandemic along with distribution of the mutations over the viral genome. Frequencies (colored by clade and normalized to 100% at each time point for 178 out of a total of 3935 tips was collected in a database called GISAID (Global Initiative on Sharing All Influenza Data, https://www.gisaid.org).

To better determine the most likely Clade in Egypt during the period between January 2020 and January 2021, we performed a phylo-geographical analysis using all available SARS-CoV-2 sequences and related global sequences from GISAID (Global Initiative on Sharing All Influenza Data, https://www.gisaid.org). These results determined the most likely clade on January 2020 is 19A and 20A. New clade 20B appear by March 2020 and 20D appear by May 2020 till January 2021 (Fig. 4). Both clades 19A and 20A were decreased by January 2021.

## Discussion

The SARS-CoV-2 outbreak was identified at Wuhan in December 2019, and the worldwide diagnosis of SARS-CoV-2 is now 21 century pandemic^18^. Globally, 111,279,860 confirmed cases of COVID-19 were reported to WHO on 23 February 2021, including 2,466,639 deaths. At the time, Egypt was ranked second high country in Africa after South Africa with 178,774 confirmed cases and 10,404 deaths. This study reveals molecular features and patterns of mutation of SARS-CoV-2 strains circulating from January 2020 to the end of January 2021 in COVID-19 Egyptian patients.

CoVs are RNA viruses with mutation-specific effects that enable rapid host replacement by mutation. The Wuhan SARS-CoV-2 strain has over 80% SARS-CoV identity and over 50% of the MERS-CoV strain that was founded in bats^19^. The SARS-CoV-2 seems to have resulted from several mutations which support the idea that virus development is a continuous process so forming new strains^20^. Two polyproteins code for 16 Nsps encoded by the viral genome. SARS-CoV-2 structural proteins are translated from single guided RNAs. Nsp functions to regulate virus replication while structural proteins are involved in binding to the receptor and virion assembly^21^. The S Protein Receptor Binding (PRB) domain selects specific mutations that improve its binding with the ACE2 receptor and improve the virus entry into the host cell^22^.

In this study SARS-CoV-2 genome sequence in COVID19 Egyptian patients were reported for high frequency mutations. ORF1ab, followed by S-gene, N gene and ORF3a, was the largest group of mutations. M, E, ORF7b, ORF7b and ORF10 have the lowest mutation rate. Of these 613 mutations, 431 synonymous mutations, 25 upstream gene mutations, 24 downstream gene mutations, 10 frameshift mutations, 6 stop mutations, and 2 conservative in-frame deletion, 2 disruptive in-frame deletion, 1 splice region mutation & synonymous mutation and 1 start loss. A similar study on 4254 SARS-CoV-2 sequences has shown that mutations are most commonly found within the ORF1a, ORF1b, as well as the S and N genes, as opposed to the ORF7b and E genes, which showed a low mutation rate frequency^23,24^. The genome's mutational frequency can be related to the increase in the infection rate of the Egyptian population and the appearance of the second wave of infection.

In the current study, 176 of 183 viral genome samples were found to be have the highest Egyptian frequency mutation D614G, where the Aspartic amino acid (D) changes to Glycine (G). The change in D614G amino acid was found both on the first and second waves in the spike region of the Egyptian GR strain. This change in amino acid was combined with a silent mutation of C241T in a non-coding region and the missense of C14408T (P214L) in ORF1b in C3037T of ORF1a. ORF1ab is transcribed into a multi-protein and then divided into 16 non-structural proteins (NSPs). The Missense mutation of C14408T and the synonymous mutation of C13536T resulting in amino acid changes (P4715L and Y4424Y) in the RNA-dependent RNA-polymerase region. One synonymous mutation of C3037T resulting in a change of amino acid p.Phe924Phe in the NSP3 region. The most frequent mutations of SARS-CoV-2 were observed in both waves of infection. The 12 top mutations in the second wave includes two mutations in N region, four mutations in ORF1ab, and two mutations in S area. Only one mutation was not present in the 1st wave of infection (RG203KR). In a further study carried out by Islam et al. 2020, 1,247 nt mutations were observed in the ORF regions and 503 of them were missense mutations^25^. NSP3, NSP4, NSP2, NSP12, and NSP5 have 120, 33, 57, 44, and 11 AA substitutions in the ORF1ab polyprotein, respectively. In the case of spike protein, 11 AA substitutions were discovered in RBD at 331 to 524 residues of S1 subunits (in Wales, the United Kingdom, Shenzhen, Hong Kong/France, Shanghai, Guangdong, Finland, and France), three of which occurred in positions 424 and 494, which comprise the receptor-binding motif (RBM). A single mutation in the S-protein in SARS-CoV-2, which was lacking in other SARS-CoV-2 strains of different geographic regions, was identified^26–29^.

Changes in ORF8 appear to be strongly linked to the adaptation of the new species, as substantial changes have been found in ORF8 during the transition from civet to human host^30^. ORF8 SARS-CoV-2 protein shares the lowest SARS-CoV homology among all viral proteins, which interacts with major histocompatibility complex molecules class I (MCH-I) and down-regulating the surface expression of MHC-I on various cells^31,32^.

Analysis of genome mutations in the first and second waves of infection compared with the global mutations in the present study has been shown to produce 4 genome mutations on an annual average and 26 on average annual mutations during Egypt's first and second waves, respectively, compared to an annual global 22,88 mutations. In the second infection wave, there is so far no specific mutation for the Egyptian samples. The presence of mutations similar to those found in other parts of the world suggests that they facilitate the adaptation of the virus to the human host. These mutations are found in NSP3, NSP6, RdRp, helicase, ORF3a, ORF8, as well as S and N proteins. These proteins are interestingly the same and have shown the highest mutation rate in our study. For the adsorption, reproduction and processing of polyproteins to replicate coronavirus, proteins are essential. In the S protein located in different domains a total of sixteen mutations were identified^33^.

Both ORF3 and ORF8 encoded proteins are type I interferon inhibitors that promote virus replication by interference with antiviral defense^34^. In the present study, nucleotide substitutions in the second wave of infection were found in 674 ORF1ab, 177 in S, 87 in N, 63 in ORF3a, 32 in ORF8, 23 in M, 20 in ORF7a, 15 in E, 8 in ORF8 and 6 in ORF6, compared to 204 mutations in the first wave of infection (131 in ORF1ab, 30 in S, 23 in N, 6 in ORF3a, 6 in ORF7a, 4 in ORF8, 2 in M, 1 in E, and 1 in ORF6). In a similar study, the changes in gene coding for N protein and ORF3a and ORF8 contributed to the epidemic's virulence, transmission and pathogens^47^. In this study, the gene codes for NSP7, NSP9, NSP10, NSP11, and ORF 7b accessory protein SARS-CoV-2 genes are not found to be mutated during the second wave of infection. Similar research study analysed the accumulation rate for the SARS-coV-2 genome over an 11-week period and found that the majority of the viral genes accumulated NSp2, NSP3, RdRp, helicase, Spike, ORF3a, ORF8 and N proteins, although with varying rates. Sixteen mutations accumulated in Spike protein, in which four mutations are located in the binding domain of the receptor. Interestingly, the number of viral proteins that did not accumulate any mutation was considered (NSP7, NSP9, NASP10, Envelop, ORF6 and ORF7b proteins)^35^. Similar to our findings, no mutations were found in NSP9, while only two amino acid substitutions were identified in NSP10^36^.

Several non-canonical structures of the nucleic acid, such as G-quadruplexes, have been shown to be essential for genome regulatory activities^37^. Although a few G-quadruplex sequences in the SARS-CoV-2 genome were determined, the inverted repetition of the genome is abundant (IRs)^38^. Two preserved SARS-CoV-2 regions are stem-loops which are designed to protect viral RNA against quick degradation and thus increase stability of the viral RNA genomes and efficiency and virulence in viral replication^39^. In the current study, to investigate the geographical distribution of SARS-CoV-2 hotspot mutations in Egyptian samples, the presence of IRs in the entire SARS-CoV-2 genome were analyzed and produced an overlay of 29 high-frequency nucleotide positions identified as hot spots based on their GISAID frequency. In SARS-COV-2 genome, potential G-quadruplex-forming sequences that regulates vital RNA syntheses are occur very rarely^40^^41^. A report showed that SARS-COV-2 genomes exhibit a CpG depletion and therefore hot-spot mutations in the SARS-COV-2 genome was important^6^.

SARS-COV-2 hot-spot mutations are significantly abundant in IR sequences and CpG islands, suggesting the SARS-COV-2 genome’s possible survival strategy and/or evolutionary benefit to the virus in either adapting to human host, modulating cellular immune response, or even increasing virulence and pathogenicity. IRs are generally very important for ssRNA genome organization^41–43^. In the present study, 29 mutations of interest were identified in the Egyptian sequences. Out of these, 18 mutations related to the variants (lineages) of interest were found in the S protein, coming from the UK B.1.1.7 lineage. Four mutations were found in the ORF1ab polyprotein, distributed in two regions coding for NSP6 (S367S), and three coded for NSP3 (T1001I),(A1798D) and (S1188L) coming from England B.1.1.7 and Brazil B.1.1.28 lineages. Three hotspot mutations were found in ORF8 (Y73C), (Q27*) and (R52I), coming from the England B.1.1.7 lineage. Three mutations of interest were observed in N protein (S235F), (T205I) and (D3L), coming from the England B.1.1.7 and South Africa B.1.351 lineages. Two mutations of interest were observed in E protein (V39L) and (P71L), coming from the England B.1.1.7 and South Africa B.1.351 lineages. The 18 mutations of interest include 12 mutations as nonsynonymous mutations, 5 as synonymous with no changes in protein sequence, and 1 of these hot-spot mutations being present at 5′ UTR. The majority of mutations change the protein sequence and can contribute to rapid modifications of their function and immunogenicity. In^42,43^, it was indicated that IRs are essential to help the virus avoid cellular immunity by organizing viral genomes. However, having these mutations of interest in IR regions can also indicate selective pressure on hairpins in certain places. Currently, COVID-19 vaccines are available in four forms: nucleic acid (mRNA and DNA), viral vector, protein subunit, and inactivated virus. Emerging SARS-CoV-2 variants, on the other hand, have raised concerns that current COVID-19 vaccines may provide less protection against Variants of Concern. Notable variants with multiple mutations in the spike protein have emerged in the United Kingdom (B.1.1.7), South Africa (B.1.351), and Brazil (P.1). The most common Variant of Concern in the second wave is B.1.1.7 (20I/501Y.V1), which has a N501Y substitution in the receptor-binding domain (RBD), a H69/V70 deletion in the N-terminal domain, and a P681H mutation in the spike protein adjacent to the furin cleavage site. This variant is associated with an increase in transmissibility. The B.1.351 variant (20H/501Y.V2) contains several mutations, including K417N, E484K, and N501Y. In the spike protein's RBD, P.1 variant (B.1.1.28.1) has K417T, E484K, and N501Y substitutions.

## Conclusion

In this paper, we analyzed SARS-COV-2 genomes from 465 Egyptian samples: 265 from first wave already deposited in the database, and new 183 sequences from the second wave. In the samples of the second wave, we detect 1115 unique mutations. The average number of mutations per samples per year increased from 4 in the first wave to 26 in the second wave. The number of Most Egyptian genomic strains sequenced in second wave of infection so far are similar to isolates from England, Brazil, and South Africa. The second wave of infection showed the relative increase of the B.1.1.1 lineages compared to B.1. Using next strain nomenclature, new clade 20B appeared in Egyptian samples by March 2020 and 20D appear by May 2020 till January 2021.

After the submission of this paper and while it was under review, we sequenced more samples as part of the continuous efforts of monitoring the changes in the SARS-COV-2 genome in the Egyptian samples. We sequenced 50 more samples from late second wave (February 2021) and 99 samples from third wave (May 2021). In these samples, we observed the emergence of the lineage C.36 (B.1.1.36) (without L452R) ranking the third place (18%) after B.1.1.1 and B.1 in the late second wave. In the third wave, C36 (with L452R) became the dominant one (49%) before B.1.1.1 and B.1. As for these new cases, there was no change in the clinical features and the death rate remained around 3%.

According to WHO, measures to combat epidemics and pandemics caused by highly pathogenic viruses may necessitate timely efforts from all or at least the majority of countries around the world. Egypt, for example, has taken unprecedented anti-epidemic measures to halt the spread of SARS-CoV2 infection.

## Material and methods

### Ethics statement

The study was permitted by the Ethics Committee of the Ministry of Health and Populations, Training and Research Sector, with number OHRP: FWA00016183 23 March 2020, IORG0005704/ IRB0000687 31 May 2020. In accordance with the principles of the 1975 Helsinki Declaration revised in 2008, the study was conducted. The study was approved by the National Institute of Cancer Ethics Committee. Before enrolling, all patients provided informed consent. After standard SARS-CoV-2 diagnostic tests were performed, the next generation sequence for SARS-CoV-2 was performed in positive samples.

Research protocol confirmatory laboratory tests have been conducted in conformity with WHO recommended. During the period of November to December 2020, all 250 samples were collected. Patients had high copy number of SARS-CoV-2 (between 1.2 × 10^4^ to 2 × 10^6^ copies/ µl) by real time PCR technique. The sequencing of QC thresholds was only achieved in 183 (172 from National Cancer Institute and 11 by the Egypt Army). There was no information available regarding the source of the isolates infection. The QIAMP VIRAL RNA mini-kit (Qiagen, Hilden, Deutschland) with internal PCR controls as instructed by the manufacturer was used with 250 to 300 µL of each nasopharyngeal swab sample for viral RNA extraction. The extracted RNA was directly used for detection of SARS-Cov2 using Genesig Real-Time PCR Detection Kit.

### Next generation sequencing of SARS-CoV-2

The RNAs collected were measured by a high-sensitivity Qubit RNA kit (Invitrogen, USA). As previously described, the entire sequence of the genome was done^44^. In brief, the genomic RNAs were retro-transcribed using the VILO-cDNA Synthesis Kit (Cat. No.11754050; Invitrogen, USA). For the preparation of the libraries, the Ion AmpliSeq Library Kit Plus (Thermo Fisher Scientific) was used. The Ion-PI-Hi-Q Sequencing 200 Kit (Thermo Fisher Scientific) PCR emulsion was used to clonally amplify the libraries. Ion PI Hi-Q Sequencing 200 Kit –Chef Kit (Thermo Fisher Scientific) of the Ion Proton Sequencer were used for the entire genome sequence.

### Data analysis

We used the pipeline for bioinformatics analysis as previously described^44^ for viral assembly and mutation calling. Briefly, the pipeline uses the Torrent Suite package (v.5.12) for alignment of the reads to the reference sequence (RefSeq; NC_045512.2), and for mutation calling. The IRMA (v0.9.3) workflow was used for de novo assembly. The de-novo assembly was compared against the reference-based assembly (based on alignment of the reads to the reference genome) to assure consistency of the results. In fact, for this target amplicon based panel, we see, as in our first paper^44^, that the reference-based assembly is enough to reconstruct the viral sequence.

As threshold of acceptance, samples with > 99% coverage and with gaps length less than 30 bps were retained for further analysis. The final successful set included 183 complete genome sequences and these were uploaded to NCBI/GISAID repositories (Supplementary File. S1).

### Lineage and phylogeny

We collected mutations and double checks for emerging strains from the UK, Brazil and South Africa, based on literature review. To assign the lineage to each sequence, the Pangolin system was used. We used MAFFT for multiple alignment computing for phylogenetic analysis (v7.450)^45^. The iqtree packages are then used to compute phylogeny, selecting the best model for nucleotide replacement with bootstrapping in order to ensure high tree topology confidence.

### Variation analysis

#### World dataset

GISAID public sequences (until 15th of January 2021) were collected and aligned to the reference viral sequence using the nucmer program^46^. The output file o is parsed to extract the variations and transform it to VCF format using in-house script. The snpEff package^47^ was then used to annotated the VCF file (snpEff_v4_5covid19_core.zip). All the VCFs were then processed to compute the frequency of each variation in the world population.

#### Egyptian dataset

To determine the characteristics of genomic variation, we analyzed the 183 whole SARS-CoV-2 genomes, collected in second wave between November 2020 and mid-January 2021. The variations (mutations) in the Egyptian genomes were examined for quality and depth. A variation is filtered out if its depth is less than 50 reads. We also checked if the variations occur in a homopolymer region or not, especially if it appears once in our dataset and not present in the world population. (Homo-polymer errors are frequent and well known sequencing errors for the Ion Torrent technology.) The final set of variations were then annotated with snpEff. Moreover, they were annotated with their frequencies in both the Egyptian and the world population.

We also analyzed the complete SARS-CoV-2 genomes of 265 samples (available on GISAID, https://www.gisaid.org) from the first wave of infection in Egypt from different institutes that were collected between March and April 2020 from 7 different institute in Egypt, namely, National Cancer Institute (n = 85), Cancer Children Hospital (n = 90), Egyptian Army (n = 36), Ain Shams Medical Institute (n = 30), Ministry of Health (n = 19), Pathogen Genomics Center, National Institute of Infectious Diseases (n = 2), National Research Center (n = 2), Vaccine Research Institute (n = 1).

## Supplementary Information

Supplementary Information.
